# Inhibiting TGF-beta signaling preserves the function of highly activated, in vitro expanded natural killer cells in AML and colon cancer models

**DOI:** 10.1371/journal.pone.0191358

**Published:** 2018-01-17

**Authors:** Folashade Otegbeye, Evelyn Ojo, Stephen Moreton, Nathan Mackowski, Dean A. Lee, Marcos de Lima, David N. Wald

**Affiliations:** 1 Department of Medicine, Division of Hematology and Oncology, University Hospitals Cleveland Medical Center, Cleveland, Ohio, United States of America; 2 Case Western Reserve University, Cleveland, Ohio, United States of America; 3 Nationwide Children’s Hospital Division of Hematology/Oncology, Columbus, Ohio, United States of America; 4 Department of Pathology, University Hospitals Cleveland Medical Center, Cleveland, Ohio, United States of America; CCAC, UNITED STATES

## Abstract

Natural killer cells harnessed from healthy individuals can be expanded ex vivo using various platforms to produce large doses for adoptive transfer into cancer patients. During such expansion, NK cells are increasingly activated and more efficient at killing cancer cells. Adoptive transfer however introduces these activated cells into a highly immunosuppressive tumor microenvironment mediated in part by excessive transforming growth factor beta (TGF-beta) from both cancer cells and their surrounding stroma. This microenvironment ultimately limits the clinical efficacy of NK cell therapy. In this study, we examined the use of a TGF-beta receptor kinase inhibitor, LY2157299, in preserving the cytotoxic function of ex vivo expanded, highly activated NK cells following sustained exposure to pathologic levels of TGF-beta in vitro and in a liver metastases model of colon cancer. Using myeloid leukemia and colon cancer cell lines, we show that the TGF-beta driven impairment of NK cell cytotoxicity is mitigated by LY2157299. We demonstrate this effect using quantitative cytotoxicity assays as well as by showing a preserved activated phenotype with high NKG2D/CD16 expression and enhanced cytokine production. In a mouse liver metastases model of colon cancer, we observed significantly improved eradication of liver metastases in mice treated with adoptive NK cells combined with LY2157299 compared with mice receiving NK cells or TGF beta inhibition alone. We propose that the therapeutic efficacy of adoptive NK cell therapy clinically will be markedly enhanced by complementary approaches targeting TGF-beta signaling in vivo.

## Introduction

The clinical development of adoptive immunotherapy with natural killer (NK) cells has been facilitated by various *ex vivo* expansion platforms that yield cell doses sufficient to achieve some clinical efficacy [1–13]. These expansion platforms typically involve co-culture of freshly isolated NK cells with irradiated antigen-presenting cells or feeder cells which are themselves sensitive to NK cell killing [4–12]. In the process of feeder cell killing, NK cells expand robustly and also acquire increasingly activated phenotypes resulting in large numbers of highly activated NK cells capable of efficient tumor killing at low effector to target ratios.

To ensure the efficacy of these highly activated NK cells in cancer therapy, it is critical that these cells maintain their cytotoxic activity in vivo. A major obstacle in this regard is that the tumor micro-environment is enriched with several immunosuppressive cytokines, one of which is transforming growth factor beta 1 (TGF-beta) [13–18]. TGF-beta is produced in excess by tumor cells themselves, as well as by regulatory T cells, myeloid derived suppressor cells (MDSCs) and other stromal cells in the tumor microenvironment. Circulating TGF-beta levels ranging from 5ng/ml to >20ng/ml have been described in both hematologic malignancies and solid tumor patients [19–23]. These levels are higher than seen in healthy volunteers and correspond with impaired cellular immunity [16–19, 24–26]. Levels below 1ng/ml have been described in the peripheral blood and bone marrow of healthy volunteers [24] while acute myeloid leukemia and myelodysplastic syndrome patients have levels ranging from 6 to 42ng/ml [21]. In a study of 45 colorectal cancer patients, Narai et al reported circulating total TGF-beta levels greater than 15ng/ml in patients with metastatic disease [20]. Those with liver metastases had the highest levels, up to 45ng/ml.

Pathologic levels of TGF beta have been shown to impair both the innate and adaptive cellular immunity of cancer patients [14,25–28]. Postulated mechanisms by which TGF-beta impairs NK cell function include down-regulated expression of activating receptors like NKG2D and CD16 (the FCγR mediating antibody-dependent, cellular cytotoxicity (ADCC)) and cytokine mediators/enzymes. It also counteracts the NK pro-survival effects of IL-2 and stimulates further proliferation of regulatory T cells. Small molecule kinase inhibitors and monoclonal antibodies targeting the TGF-beta receptor have been explored as a means of enhancing cellular immune response pre-clinically [15,27–29]. There is at least one active clinical trial exploring the combination of a TGF-beta receptor inhibitor, LY2157299 (Galunisertib, Eli Lilly) with the PD-1 monoclonal antibody Nivolumab, with a goal of enhancing the liberated T-cell response.

In the process of developing allogeneic adoptive transfer of NK cells as a therapeutic strategy against various malignancies, we have adopted *ex vivo* expansion of NK cells using antigen-presenting feeder cells. In the process of generating large cell yields during expansion, the resulting NK cells are also significantly more activated and better efficient at killing both liquid and solid tumor targets. Our hypothesis is that these highly activated NK cells will again have limited clinical efficacy in vivo after being continually exposed to the immunosuppressive, TGF-beta rich microenvironment of cancer patients following adoptive transfer. This will limit the clinical efficacy of such therapeutic endeavors. In this study we explored inhibiting TGF-beta signaling as a strategy to preserve and/or enhance the cytolytic efficacy of ex vivo expanded, highly activated NK cells in the TGF-beta rich milieu of myeloid leukemia and metastatic colon cancer.

## Materials and methods

### NK cell culture and activation

Procurement of peripheral blood samples from healthy volunteers with written informed consent for research use. The Institutional Review Board (IRB) of University Hospitals Cleveland Medical Center approved the procurement and use of blood samples for this study. Peripheral blood mononuclear cells (MNCs) were separated into buffy coats following density gradient centrifugation of whole blood over Ficoll-Paque Plus (GE Healthcare Life Sciences). MNCs were subjected to CD3 depletion followed by CD56 enrichment using MACS human CD3 depletion and human NK cell enrichment kits respectively according to the manufacturer’s instructions (Miltenyi Biotech). The CD3-, CD56+ NK cells (>98% purity confirmed by flow cytometry) were either incubated overnight in media supplemented with IL-2 (GoldBio) for next day assays or were expanded over 14 days in co-culture with irradiated feeder cells (K562-mbIL21) and IL-2 (50U/mL) as described by Somanchi et al [9]. NK cells were subsequently maintained in IL-2 supplemented media either alone or in combination with human TGF-beta 1 (Cell Signaling) at 5-10ng/ml for up to 96 hours. The base medium was RPMI 1640 + Glutamine 2.05mM (HyClone), 10% Cosmic Calf Serum (HyClone) and 1% penicillin-streptomycin.

Immediately prior to all in vitro assays, NK cells were centrifuged out of cell culture and resuspended in fresh media to remove continued IL2, TGF beta or LY2157299 exposure of the NK cells and their co-cultured targets.

### Cytotoxicity assay

NK cell cytotoxicity against various cancer cell lines was obtained using a quantitative flow cytometry assay. Cancer cells used were a myeloid leukemia (HL60), and colon cancer cell lines (HCT116 and HT29) cultured in the base medium described above and all were obtained from ATCC. Target cells were labeled with Calcein-AM (Calbiochem) and then co-incubated with NK cells at varying effector (NK cell) to target ratios. 10,000 target cells were used per triplicate well for all experiments. At the end of 4 hours incubation, the number of live CAM-labeled target cells per 100uL of cell suspension was quantified by rapid flow cytometry with an Attune NxT flow cytometer (Thermo Fisher Scientific). Cytotoxicity results are expressed as the proportion of Calcein-AM (CAM) bright cells.

NK Cell Cytotoxicity = (Number of CAM bright cells in a target cell alone well *minus* Number of CAM bright cells in NK + target co-culture) *divided by* Number of CAM bright cells in a target cell alone well.

### NK cell phenotyping

In cell culture assays NK cells were phenotyped by flow cytometry for CD16 and NKG2D at various time points using anti-CD56-APC (BD-Biosciences), and anti-CD16-PE (BD-Biosciences). Antibody staining of single cell-suspensions was performed by manufacturer’s instructions/protocol. Stained cells were analyzed by flow cytometry.

### ELISA assays

Quantification of human IFN-gamma, TNF-alpha, Perforin and Granzyme B was performed using commercially available ELISA kits (R&D signaling). For IFN-gamma and TNF-alpha quantification, cell-free supernatants were collected after 4h of co-incubation at 37°C of 40,000 NK cells and 10,000 colon cancer cells (HT29). For Perforin and Granzyme B quantification, cell-free supernatants were collected after 2h of co-incubation of 10^6^ NK cells and 10^6^ HL60 cells. Results shown are the means of triplicate wells ± standard deviation measurements. Active human TGF-beta levels were measured in the serum of human colon cancer murine xenografts following the manufacturer’s protocol (R&D signaling). To prepare mouse sera for the TGF-beta assay, cardiac blood collected immediately following mouse euthanasia was transferred to pre-cooled micro-centrifuge tubes containing EDTA and centrifuged for 30 min at 1,500g at 4°C. The supernatant plasma was collected and stored in micro-centrifuge tubes at -80°C. Samples were thawed at room temperature on the day of TGF beta assay.

### Colon cancer liver metastases mouse model and human NK cell adoptive transfer

100,000 HCT116 cells were surgically implanted into NSG mouse spleens (following hemi-splenectomy); 4 groups, 3 mice/group. The control group received tail vein injections of 3% FBS/PBS and twice daily oral gavage of inhibitor carrier. A TGF-B inhibitor only group received tail vein injections of 3% FBS/PBS and twice daily oral gavage of LY2157299 (TGF-beta inhibitor) at a dose of 75mg/kg twice daily for 2 weeks. A third group (NK alone) received 5 x 10^6^ NK cells each (tail vein), weekly for two weeks, starting 10 days after hemi-splenectomy. The fourth group (NK + TGF-B inhibitor) received 5 x 10^6^ NK cells each (tail vein), weekly for two weeks, starting 10 days post-op and the TGF-beta inhibitor LY2157299 by oral gavage twice daily at 75mg/kg for two weeks. Mice receiving NK cells also received IL2 (75,000U IP) three times a week (MWF) for two weeks. The vehicle for LY2157299 was constituted per manufacturer (Eli Lilly) instructions [30]. The vehicle for mouse injections (cells and IL2) was sterile filtered PBS with 3% calf serum.

This study was carried out in strict accordance with recommendations in the Guide for the Care and Use of Laboratory Animals of the National Institutes of Health. Mouse surgeries were performed under ketamine/xylazine anesthesia, and all efforts were made to minimize suffering in the peri-operative period and during oral gavaging including post-operative analgesia per institutional guidelines. All mice were euthanized at a pre-determined end point 1 week after the second NK cell infusion in treated mice. Euthanasia was performed using carbon dioxide asphyxiation followed by cervical dislocation. All procedures used in mouse care, surgeries and euthanasia for the purpose of this study were approved by the Institutional Animal Care and Use Committee of Case Western Reserve University.

### Statistical analysis

All continuous measurements (NK cell expression of CD16, NKG2D, and change in cytotoxicity during in vitro studies were compared using the student T-test between two groups (control NK cells were reference sample). Tumor burden measurements computed from imaging in colon cancer liver metastasis xenograft was compared using ANOVA among groups (≥ 3). P-values demonstrated are at significance levels of 0.05 for two-tailed hypotheses. Chart indicators for p-values are * if <0.05; ** if ≤0.01, *** if ≤0.001, **** if ≤0.0001 and ns if ≥0.05

## Results

### Ex vivo expansion of NK cells leads to increased cytotoxic activity against leukemia and colon cancer

**Fig 1:**
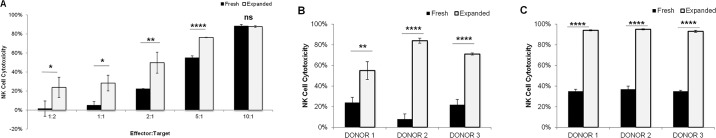
Expanded NK cells demonstrate increased cytotoxicity against HCT116 and HT29 cells as compared to fresh, IL-2 activated cells. The indicated NK cells were assessed for cytotoxic activity against target cancer cells using a calcein AM flow cytometry assay following 4 hours co-incubation. (A) Percentage cell death of HCT116 cells induced by NK cells at the indicated Effector:Target cell ratios. (B and C) Percentage HT29 cell death induced by NK cells from 3 individual healthy donors at Effector:Target ratios of 1:1 (B) and 4:1 (C). Bars represent data using IL2-activated NK cells (FRESH) and following expansion of NK cells from the same donor (EXPANDED). *p<0.05; **p≤0.01; ***p≤0.001; ****p≤0.0001; ns p≥0.05.

Ex vivo expanded NK cells exhibit increased cytotoxic activity against colon cancer and myeloid leukemia cells. We compared the cytotoxic function of freshly isolated NK cells which were overnight activated in IL2 with NK cells from the same donor that were expanded for 2 weeks using irradiated K562mIL21 feeder cells. The expanded NK cells were more efficient at killing both colon cancer and myeloid leukemia cells. For example at a 1:2 NK cell to target cell ratio, there was 24% killing of HCT116 cells as compared to 2% killing with fresh NK cells; p = 0.043 (Fig 1A). Overnight-activated NK cells required an effector to target ratio of 5:1 to achieve 50% killing while expanded cells achieved this at 2:1 ratio. In addition to HCT116 cells, expanded NK cells also exhibited high cytotoxicity on HT29 cells (Fig 1B). At a 1:1 ratio using 3 different NK cell donors, the expanded NK cells killed 45–86% of HT29 cells (Fig 1B). This cytotoxic effect was significantly better than the overnight activated NK cells which killed 4–28% of the HT29 cells. Again, expanded NK cells demonstrated superior killing efficiency at 4:1 ratios, with >90% killing across all donors at a 4:1 ratio as compared to 29%-40% using fresh NK cells (Fig 1C).

### Exposure to pathologic TGF-beta levels impairs expanded NK cell function in a time-dependent manner

**Fig 2:**
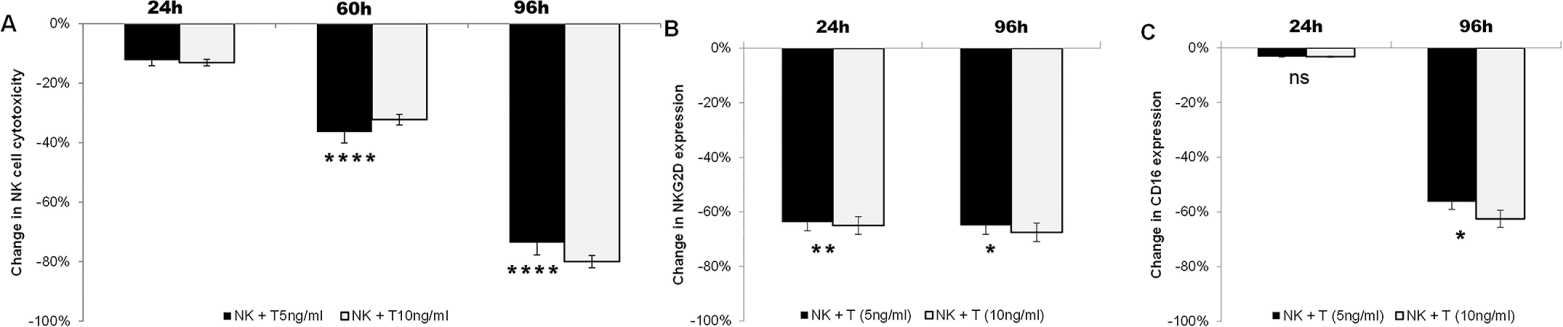
Sustained exposure to pathologic levels of TGF-beta impairs the function of highly activated, expanded NK cells. After 2 weeks of expansion, NK cells were maintained in culture supplemented with 200U/ml IL2 with or without TGF-beta at 5ng/ml and 10ng/ml. (A) Change in cytotoxicity of expanded NK cells against HL60 cells (4:1 ratio) following exposure to indicated doses of TGF-beta after 24h, 60h and 96h. Change presented is in comparison to NK cells maintained without TGF-beta. (B and C) Change in activated phenotype following 24h and 96h exposure to the indicated doses of TGF-beta as compared to control NK cells. Change in proportion of NK cells expressing NKG2D (B) and CD16 (C) are shown. T: TGF-beta ligand. *p<0.05; **p≤0.01; ****p≤0.0001; ns p≥0.05.

At TGF-beta levels similar to that found in AML patients (5ng/ml), NK cell killing of the myeloid leukemia cell line HL60 was progressively impaired at 24 hours (11–14% decrease, p = 0.002), 72 hours (33–41% decrease, p<0.0001) and 96 hours exposure (70–78% decrease, p<0.0001) (Fig 2A). This reduced cytotoxic activity correlated with an NK cell receptor phenotype consisted with less active NK cells. For example, there was a 65–68% decline in NKG2D expression as early as 24 hours in the NK cells (Fig 2B; p = 0.005). CD16 expression did not change in the first 24 hours of exposure but decreased by 56% at 96 hours (Fig 2C; p = 0.037). There was no appreciable difference by increasing the TGF-beta ligand concentration from 5ng/ml to 10ng/ml (Fig 2B and 2C).

### TGF-beta inhibition maintains the function of expanded NK cells despite sustained exposure to pathologic TGF-beta levels

**Fig 3:**
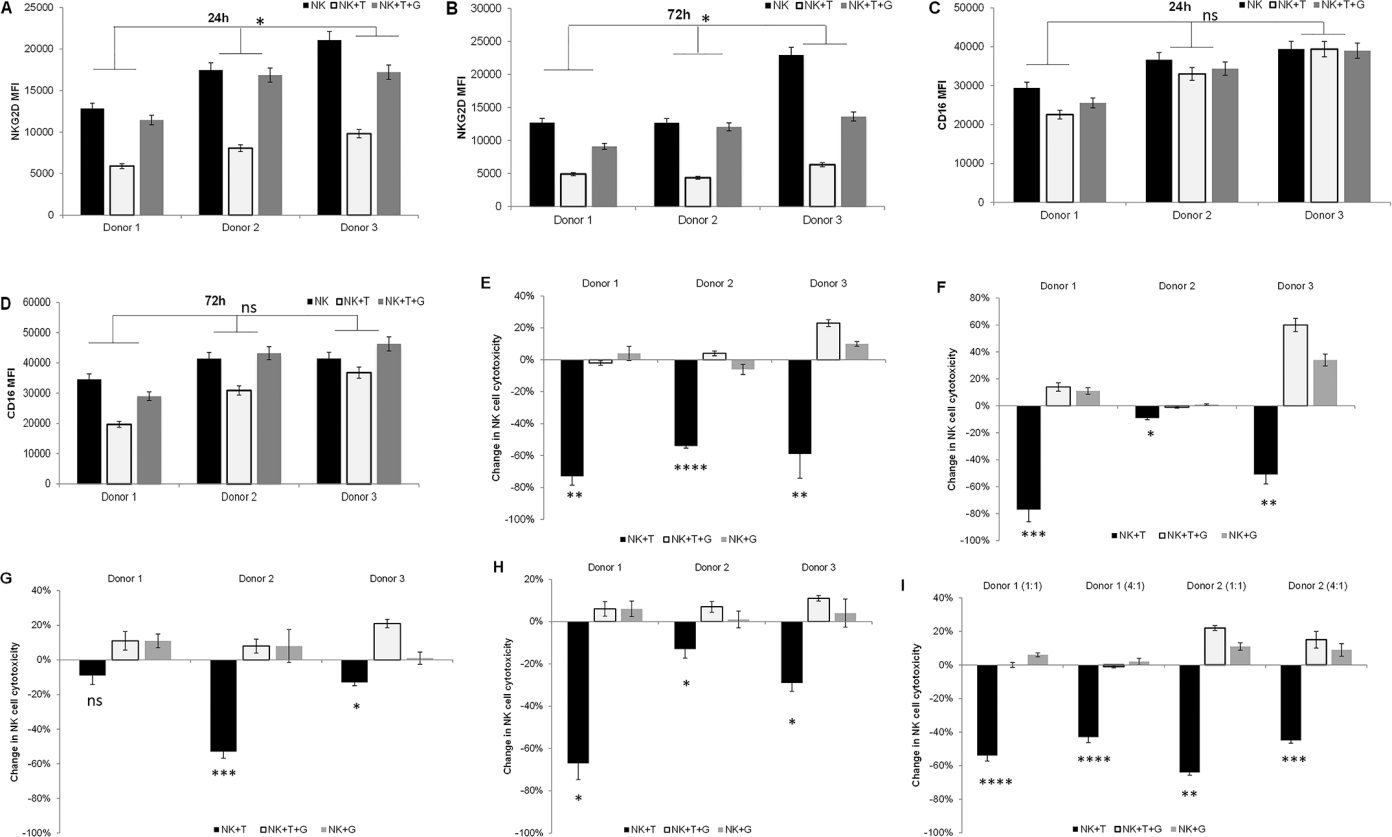
Inhibiting TGF beta signaling using the small molecule kinase inhibitor LY2157299 preserves the cytotoxic function of expanded NK cells, even after sustained exposure to pathologic levels of TGF-beta. After 2 weeks of expansion, NK cells were maintained in culture supplemented with 200U/ml IL2 alone or in combination with TGF-beta 1 (5ng/ml) and/or LY2157299 (5uM) for the indicated times and tested by flow cytometry and cytotoxic assays. (A, B, C and D) Mean fluorescence intensity (MFI) of NKG2D after 24h (A) and 72h (B); and for CD16 after 24h (C) and 72h (D). (E, F, G and H) Change in cytotoxicity compared to control NK cells (not treated with TGF-beta or LY2167299) against HT29 cells at 24h (E) and 72h (F); and against HCT116 at 24h (G) and 72h (H). Individual results from three different donors using NK to target ratios of 1:1 are presented in each figure. (I) Similar 72 hour results using NK cells obtained from two donors is shown against HL60 cells using NK to HL60 ratios of 1:1 and 4:1. T: TGF-beta; G: LY2157299. *p<0.05; **p≤0.01; ***p≤0.001; ****p≤0.0001; ns p≥0.05.

We assessed the phenotype and cytotoxic function of ex vivo expanded NK cells treated with TGF-beta alone or in combination with LY2157299, a clinically used oral small molecule kinase inhibitor of TGF-beta receptor 1. Despite continued exposure to TGF-beta, the ‘activated’ NK cell phenotype consisting of NKG2D+CD16 bright NK cells was preserved by the addition of LY2157299 (Fig 3A to 3D). NKG2D expression was decreased by approximately 53% at 24 hours and 61%-72% by 72 hours with TGFbeta alone; p = 0.025 and 0.035 respectively (Fig 3A and 3B). This change was significantly ameliorated at 24 and 72 hours after treatment with the addition of LY2157299 (p = 0.029 and 0.012 respectively). A change in CD16 expression was not evident at 24 hours (Fig 3C) but was decreased by 11%-43% by 72 hours (Fig 3D); p = 0.071. These changes were again prevented by the addition of LY2157299.

We also measured killing of colon cancer and leukemia cell lines by the ex vivo expanded NK cells in the presence of TGFbeta and/or LY2157299. At a NK to target ratio of 1:1, TGF-beta exposure resulted in a 54%-73% decrease in killing of HT29 cells (Fig 3E) after 24 hours and 9–77% after 72 hours (Fig 3F) The addition of LY2157299 preserved the cytotoxic function of NK cells in the presence of TGF-beta ligand, and a modest gain of function in cells from one donor. Changes in NK cell cytotoxicty after 24 hours exposure to TGFbeta were more modest against HCT116; -9%, -53%; and -13% in individual donors (Fig 3G). After 72 hours TGF-beta exposure, a more marked decrease in cytotoxic activity against HCT116 was noted; -67%, -13% and -29% (Fig 3H).

Against HL-60 cells, there was a 54%-64% decrease in NK cell mediated killing after 72 hours with TGF-beta exposure at a 1:1 NK cell to target ratio (Fig 3I). Increasing the NK cell ratio to 4:1 was still associated with roughly a 40% decrease in cytotoxic function. Again, the addition of the TGF-beta inhibitor LY2157299 preserved the high cytotoxic function of these NK cells.

**Fig 4:**
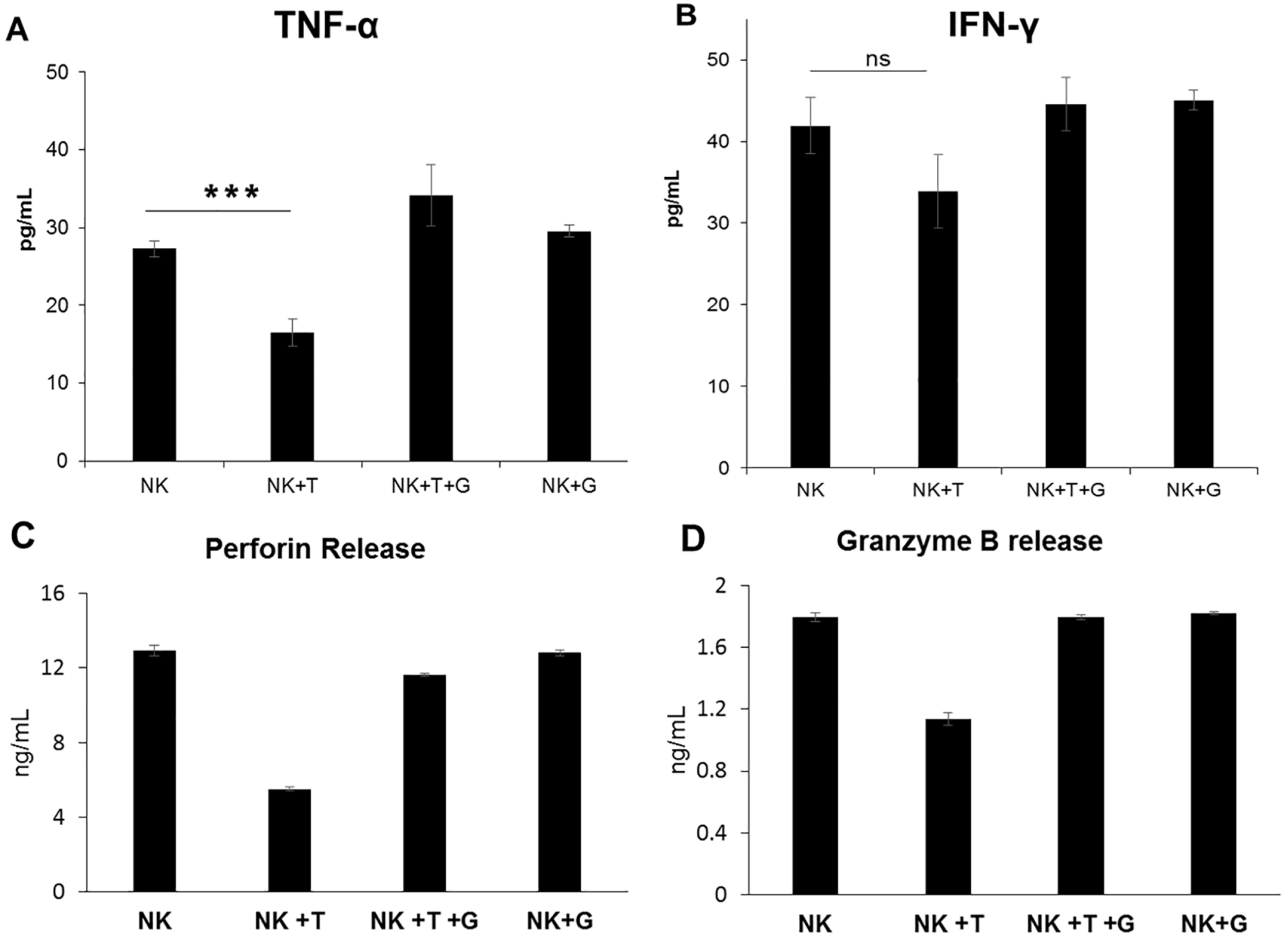
TGF-beta signaling impairs the production of TNF-alpha, IFN-gamma, Perforin and Granzyme B by ex vivo expanded NK cells. Functional assay of expanded NK cells after 72h exposure to 5ng/ml TGF-beta ligand by measuring release of TNF-alpha and IFN-gamma into the supernatant at the end of a 4hr cytotoxicity assay. ELISA quantification of TNF-alpha (A) and IFN-gamma (B) in the supernatant of 40,000 NK cells co-cultured with 10,000 HT29 cells/well. ELISA quantification of perforin (C) and granzyme B (D) release after 2 hours of co-culturing 10^6^ NK cells with 10^6^ HL60 cells are presented. All assays presented are results of triplicates. T: TGF-beta; G: LY2157299. ***p<0.001; **** p≤0.0001; ns p≥0.05.

The production of TNF-alpha and IFN-gamma by these otherwise highly activated NK cells was also significantly impaired following TGF-beta exposure. During co-culture with HT29 cells, NK cells previously incubated with TGF-beta released 40% less TNF-alpha; p = 0.0007 (Fig 4A) and 19% less IFN-gamma than control NK cells; p = 0.069 (Fig 4B). Compared with control NK cells, TGF-beta treated NK cells maintained in culture with LY2157299 showed a slight trend to increased production of TNF-alpha (1.25-fold, p = 0.05) and IFN-gamma (1.06-fold, p = 0.4). In addition to functional cytotoxicity assays, we measured the release of Perforin and Granzyme B by NK cells co-cultured with a leukemia target cell line (HL60). We again noted a decrease in the levels of both perforin (by 57%; p<0.00001) and granzyme B (by 38%; p<0.0001) by NK cells exposed to TGF beta ligand (Fig 4C and 4D)

### TGF-beta inhibition results in superior tumor eradication by highly activated NK cells in a metastatic colon cancer mouse model

**Fig 5:**
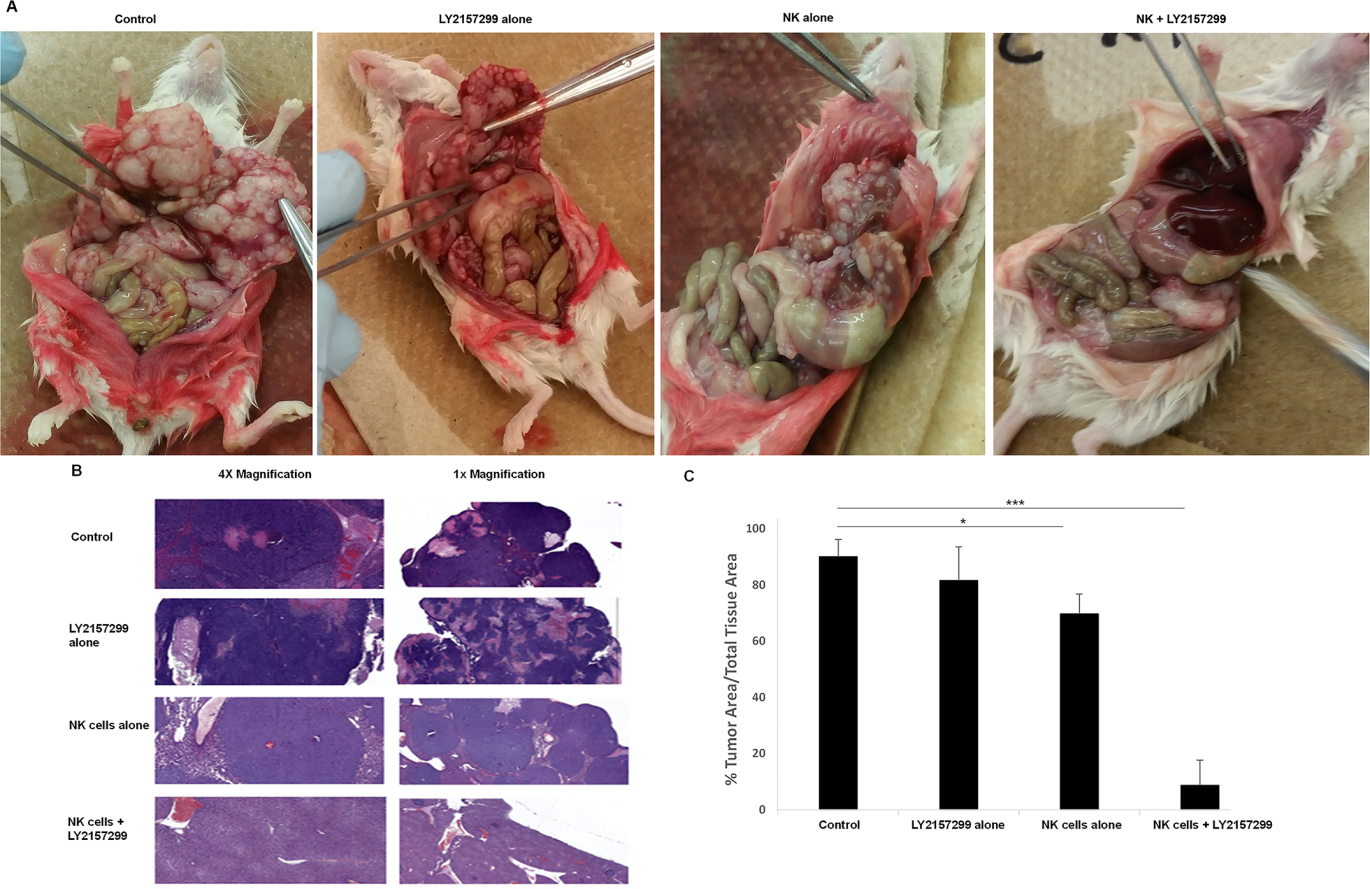
TGF-beta inhibition enhances activated NK cell function in a colon cancer model of liver metastases. A liver metastases model of colon cancer xenograft using HCT116 cells was established. All mice were autopsied at 32 days after cell injection and H&E sections were obtained. Tumor burden on H&E sections was measured for each mouse liver. (A) Autopsy findings of gross liver morphology in one representative mouse from each of the four treatment groups. (B) Representative light microscopy of H&E stained liver sections. (C) Average tumor burden in H&E stained liver sections (N = 3 mice/group) was quantified using the VENTANA digital Image viewing software. * p <0.05; *** p ≤0.001.

We examined the therapeutic efficacy of ex vivo expanded NK cells in an immunodeficient mouse (NSG) model of colon cancer liver metastasis using HCT116 cells. Four weeks after splenic implantation of HCT116 cells all mice were euthanized. At autopsy, control and LY2157299 alone mice had hemorrhagic ascites and grossly enlarged livers, riddled with metastatic deposits without evidence of normal liver tissue (Fig 5A); panel 1 and 2. Mice treated with NK cells alone also had gross evidence of liver metastases however nodule burden was less and there was some morphologic evidence of normal liver architecture (Fig 5A); panel 3. Mice that received NK cells and LY2157299 had mostly normal liver morphology with rare gross nodules (Fig 5A); panel 4. H&E stains of liver sections for all 3 mice per group were consistent with gross anatomy (Fig 5B and 5C). Quantification of the tumor burden showed a 25% decrease in mice treated with NK cells alone (p<0.05) and by approximately 90% in those treated with NK cells and LY2157299; p<0.001 (Fig 5C). Of note, circulating human TGF-beta levels (active) in the mice of all treatment groups was an average of 10.98pg/ml + 1.736pg/ml. These levels are comparable to serum levels of active TGF-beta in colorectal cancer patients. [20]

### TGF-beta inhibition enhances NK cell infiltration into liver tissue in colon cancer metastasis model

**Fig 6:**
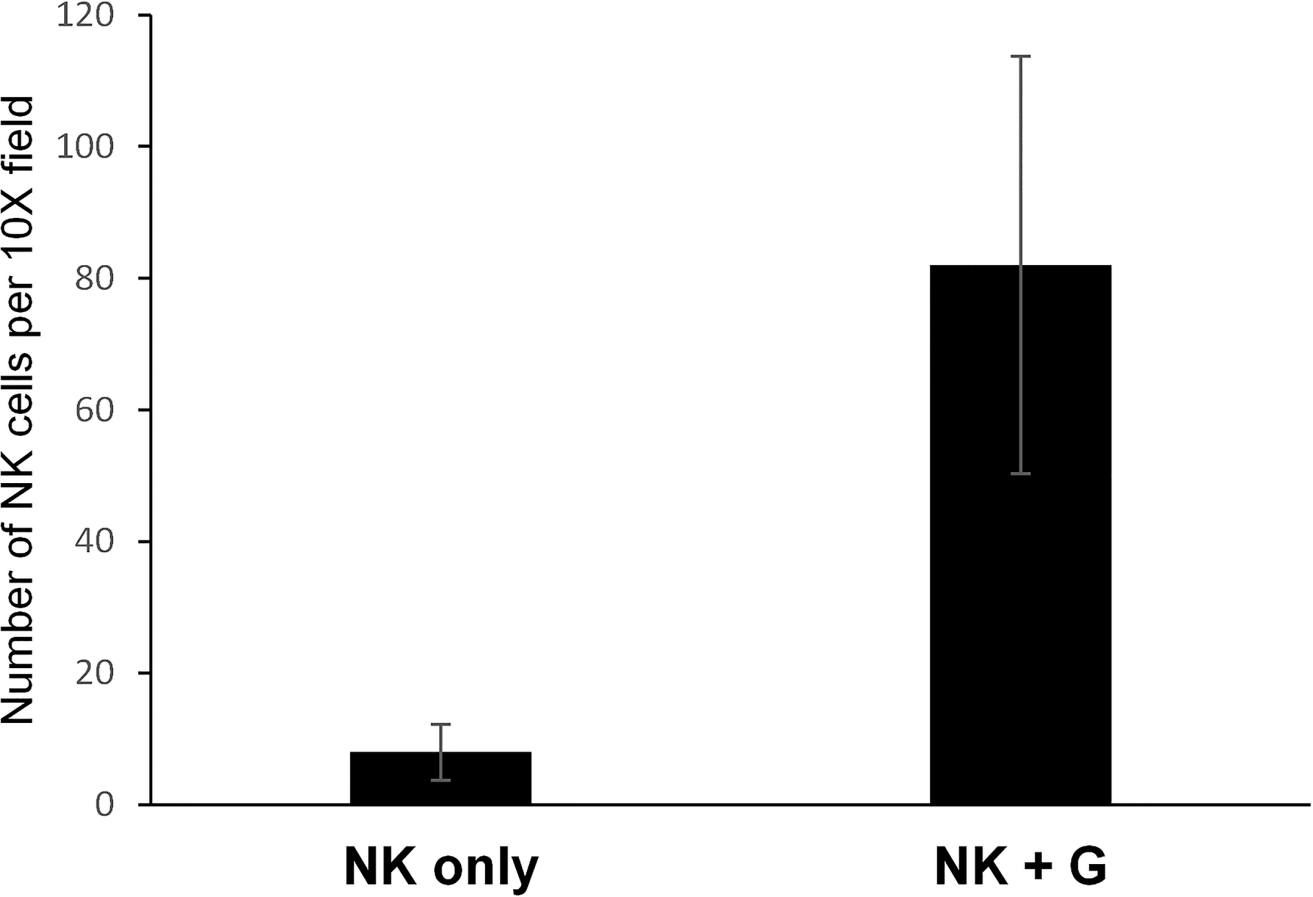
Robust NK cell infiltration into liver tissue observed in mice who received TGF-beta inhibition in addition to NK cell infusion. FFPE sections of liver tissue from mice with metastatic colon cancer were subjected to immunohistochemistry with human specific CD45. Manual counts of CD45 positive cells (NK cells) was done in four representative 10X fields using the VENTANA digital image viewing software. Mice that received NK cells alone (NK only) and NK cells with LY2157299 (NK+G).

In our metastatic colon cancer xenograft, we examined FFPE liver sections for NK cell infiltration using immunohistochemistry for human specific CD45 antibody. Mice that received NK cells in addition to LY2157299 had an average of 82 infiltrating NK cells per 10X field compared to an average of 8 cells in similar fields in mice that did not receive TGF-beta inhibition; p = 0.0036 (Fig 6). Of note, mice were necropsied 14 days after the last of two NK cell infusions and 9 days after the last dose of LY2157299. Pictures of representative sections are provided in S1 Fig.

## Discussion

Despite the promise of NK cell therapy, the approach has been limited due to several factors including challenges in generating sufficient levels of NK cells for infusion as well as difficulties in maintaining a high level of activity in vivo of the infused cells. The development of robust feeder cell lines for ex vivo NK cell expansion has enabled the production of massive doses of NK cells [4–12]. These strategies include the use of antigen presenting cells as stimuli for NK cell activation and subsequent proliferation. As an example, the K562-mbIL21 cells used in our study are K562 cells transduced to express membrane-bound interleukin 21 and 4-1BB ligand resulting in up to 21,000 fold proliferation of NK cells after three weeks co-culture [9]. Besides a high level of expansion, an added advantage of ex vivo expansion is that the resulting NK cells are also significantly activated compared to resting NK cells and are more efficient at killing cancer targets. Consequently, it becomes even more pertinent to ensure that these highly activated killer cells remain protected from the immunosuppressive tumor microenvironment after infusion. Studies have shown that the tumor microenvironment produces high levels of factors such as IL-10 and TGFbeta leading to NK cell dysfunction [12–23,25].

In this report we show that TGF-beta inhibition with a clinically used agent, LY2157299, can effectively mitigate TGF-beta-mediated NK cell dysfunction using in vitro and in vivo model systems of leukemia and colon cancer. We have demonstrated that the marked impairment in function of highly activated NK cells starting from 24 hours exposure to a TGF-beta rich milieu correlates with a significant decrease in expression of the activating receptors NKG2D and CD16. Inhibiting TGF-beta signaling in this setting maintained the activated phenotype of these NK cells and resulted in more effective NK cell-mediated eradication of colon cancer liver metastases in a mouse model.

LY2157299 and TEW-7197 are the only two small molecule inhibitors of TGF-beta receptor 1 currently in clinical trials for various malignancies, particularly advanced stage, solid tumors like pancreatic, prostate and hepatocellular carcinoma [30–32]. These trials are either ongoing or recently completed with results not yet published. For our current research we chose the compound LY2157299 as it is further along in clinical development and the safety/pharmacokinetic profile has been well characterized in the aforementioned cancer trials as well as in healthy individuals (NCT01965808). There are now clinical trials aimed at enhancing the effector T cell response from checkpoint blockade via TGF-beta inhibition by combining LY2157299 with Nivolumab in various advanced solid tumors (NCT02423343) and with Durvalumab in pancreatic cancer (NCT02734160). There are also ongoing trials of adoptively transferred NK cells predominantly for hematologic malignancies without complementary TGF beta inhibition. From our current study, we propose complementing adoptive transfer of NK cells with such TGF-beta inhibiting agents to maintain these cells in their highly activated state and ensure better clinical efficacy. We have focused now on colon cancer and leukemia models; in future studies we will assess the general applicability of this strategy for other tumor types.

## Supporting information

S1 Table Tables comparing cytotoxic activity of fresh, IL2 activated versus feeder-expanded NK cells. (PDF)

S2 Table Tables showing functional analysis of NK cells following exposure to TGF-beta 1 ligand. (PDF)

S3 Table Functional analysis of NK cells in vitro with or without inhibition of TGF beta signaling. (PDF)

S4 Table Comparisons of TNF-alpha, IFN-gamma, Perforin and Granzyme release. (PDF)

S1 Fig Representative images of colon cancer metastasis xenograft showing CD45 IHC of liver FFPE sections staining for human NK cells. (PDF)

S2 Fig Dose titration of LY2157299 for preservation of NK cell cytotoxic function. (PDF)

## References

[1] Geller MA, Miller JS. Use of allogeneic NK cells for cancer immunotherapy. Immunotherapy. 2011 12; 3(12): 1445–1459. doi: 10.2217/imt.11.131 22091681 10.2217/imt.11.131 PMC3292871

[2] Cheng M, Vhen Y, Xiao W, Sun R, Tian Z. NK cell-based immunotherapy for malignant diseases. Cell Mol Immunol. 2013 5;10(3):230–52. doi: 10.1038/cmi.2013.10 23604045 10.1038/cmi.2013.10 PMC4076738

[3] Knorr DA, Bachanova V, Verneris MR, Miller JS. Clinical utility of natural killer cells in cancer therapy and transplantation. Semin Immunol. 2014 4;26(2):161–72 doi: 10.1016/j.smim.2014.02.002 24618042 10.1016/j.smim.2014.02.002 PMC3984606

[4] Cho D, Campana D. Expansion and activation of natural killer cells for cancer immunotherapy. Korean J Lab Med. 2009 4;29(2):89–96 doi: 10.3343/kjlm.2009.29.2.89 19411773 10.3343/kjlm.2009.29.2.89 PMC2771620

[5] Childs RW, Berg M. Bringing natural killer cells to the clinic: ex vivo manipulation. Hematology Am Soc Hematol Educ Program. 2013;2013:234–46. doi: 10.1182/asheducation-2013.1.234 24319186 10.1182/asheducation-2013.1.234 PMC6610030

[6] Tomchuck S, Leung W, Dallas M. Isolation, expansion and function of cord blood natural killer cells. J Immunol May 1, 2013, 190 (1 Supplement) 69.51;

[7] Szmania S, Lapteva N, Garg T, Greenway A, Lingo J, Nair B et al Ex vivo-expanded natural killer cells demonstrate robust proliferation in vivo in high-risk relapsed multiple myeloma patients. J Immunother. 2015 1;38(1):24–36. doi: 10.1097/CJI.0000000000000059 25415285 10.1097/CJI.0000000000000059 PMC4352951

[8] Spanholtz J, Preijers F, Tordoir M, Trilsbeek C, Paardekooper J, de Witte T et al Clinical-grade generation of active NK cells from cord blood hematopoietic progenitor cells for immunotherapy using a closed-system culture process. PLoS One. 2011;6(6):e20740 doi: 10.1371/journal.pone.0020740 21698239 10.1371/journal.pone.0020740 PMC3116834

[9] Somanchi SS, Senyukov VV, Denman CJ, Lee DA. Expansion, purification, and functional assessment of human peripheral blood NK cells. J Vis Exp. 2011 2 2;(48). 10.3791/2540 PMC3180743 21339714

[10] Shah N, Martin-Antonio B, Yang H, Ku S, Lee DA, Cooper LJ et al Antigen presenting cell-mediated expansion of human umbilical cord blood yields log-scale expansion of natural killer cells with anti-myeloma activity. PloS One 2013 10 18;8(10): e76781 doi: 10.1371/journal.pone.0076781 24204673 10.1371/journal.pone.0076781 PMC3800010

[11] Lapteva N, Durett AG, Sun J, Rollins LA, Huye LL, Fang J et al Large-scale ex vivo expansion and characterization of natural killer cells for clinical applications. Cytotherapy. 2012 10;14(9):1131–43. doi: 10.3109/14653249.2012.700767 22900959 10.3109/14653249.2012.700767 PMC4787300

[12] Kang L, Voskinarian-Berse V, Law E, Reddin T, Bhatia M, Hariri A et al Characterization and ex vivo Expansion of Human Placenta-Derived Natural Killer Cells for Cancer Immunotherapy. Front Immunol. 2013 5 1;4:101 doi: 10.3389/fimmu.2013.00101 23641243 10.3389/fimmu.2013.00101 PMC3640206

[13] Bierie B, Moses HL. Tumour microenvironment: TGFbeta: the molecular Jekyll and Hyde of cancer. Nat Rev Cancer. 2006 7;6(7):506–20 doi: 10.1038/nrc1926 16794634 10.1038/nrc1926

[14] Bellone G, Aste-Amezaga M, Trinchieri G, Rodeck U. Regulation of NK cell functions by TGF-beta 1. J Immunol. 1995 8 1;155(3):1066–73 7636180

[15] Bergmann L, Schui DK, Brieger J, Weidmann E, Mitrou PS, Hoelzer D. The inhibition of lymphokine-activated killer cells in acute myeloblastic leukemia is mediated by transforming growth factor-beta 1. Exp Hematol. 1995 12;23(14):1574–80 8542949

[16] Konjevic G, Jurisic V, Jovic V, Vuletic A, Mirjacic Martinovic K, Radenkovic S et al Investigation of NK cell function and their modulation in different malignancies. Immunol Res. 2012 4;52 (1–2):139–56 doi: 10.1007/s12026-012-8285-7 22442005 10.1007/s12026-012-8285-7

[17] Lee HM, Kim K-S, Kim J. A comparative study of the effects of inhibitory cytokines on human natural killer cells and the mechanistic features of transforming growth factor-beta. Cell Immunol. 2014 7;290(1):52–61. doi: 10.1016/j.cellimm.2014.05.001 24879062 10.1016/j.cellimm.2014.05.001

[18] Baginska J, Viry E, Paggetti J, Medves S, Berchem G, Moussay E et al The critical role of the tumor microenvironment in shaping natural killer cell-mediated anti-tumor immunity. Front Immunol. 2013 12 25;4:490 doi: 10.3389/fimmu.2013.00490 24400010 10.3389/fimmu.2013.00490 PMC3872331

[19] Lee JC, Lee KM, Kim DW, Heo DS. Elevated TGF-beta1 secretion and down-modulation of NKG2D underlies impaired NK cytotoxicity in cancer patients. J Immunol. 2004 6 15;172(12):7335–40 15187109 10.4049/jimmunol.172.12.7335

[20] Narai S, Watanabe M, Hasegawa H, Nishibori H, Endo T, Kubota T et al Significance of transforming growth factor beta1 as a new tumor marker for colorectal cancer. Int J Cancer. 2002 2 1;97(4):508–11 11802214 10.1002/ijc.1631

[21] Akiyama T, Matsunaga T, Terui T, Miyanishi K, Tanaka I, Sato T et al Involvement of transforming growth factor-B and thrombopoietin in the pathogenesis of myelodysplastic syndrome with myelofibrosis. Leukemia. 2005 9;19(9):1558–66 doi: 10.1038/sj.leu.2403875 16034467 10.1038/sj.leu.2403875

[22] Blobe GC, Dong M. Role of transforming growth factor-beta in hematological malignancies. Blood. 2006 6 15;107(12):4589–96. doi: 10.1182/blood-2005-10-4169 16484590 10.1182/blood-2005-10-4169 PMC1895802

[23] Hong C, Muller L, Whiteside TL, Boyiadzis M. Plasma exosomes as markers of therapeutic response in patients with acute myeloid leukemia. Front Immunol. 2014 4 10;5:160 doi: 10.3389/fimmu.2014.00160 24782865 10.3389/fimmu.2014.00160 PMC3989594

[24] Hirayama Y, Sakamaki S, Tsuji Y, Sagawa T, Chiba H, Matsunaga T et al Thrombopoietin concentrations in peripheral blood correlated with platelet numbers in two patients with thrombocytopenia by chronic graft-versus-host disease. Am Journal of Hematology. (2003)73:285–289 10.1002/ajh.10345 12879435

[25] Dasgupta S, Bhattacharya-Chatterjee M, O’Malley BW, Chatterjee SK. Inhibition of NK cell activity through TGF-B1 by down-regulation of NKG2D in a murine model of head and neck cancer. J Immunol. 2005 10 15;175(8):5541–50. 16210663 10.4049/jimmunol.175.8.5541

[26] Trotta R, Dal Col J, Yu J, Ciarlariello D, Thomas B, Zhang X et al TGF-beta utilizes SMAD3 to inhibit CD16-mediated IFN-gamma production and antibody-dependent cellular cytotoxicity in human NK cells. J Immunol. 2008 9 15;181(6):3784–92. 18768831 10.4049/jimmunol.181.6.3784 PMC2924753

[27] Chretien AS, Le Roy A, Vey N, Prebet T, Blaise D, Fauriat C et al Cancer-Induced Alterations of NK-Mediated Target Recognition: Current and Investigational Pharmacological Strategies Aiming at Restoring NK Mediated Anti-Tumor Activity. Front Immunol. 2014 3 24;5:122 doi: 10.3389/fimmu.2014.00122 24715892 10.3389/fimmu.2014.00122 PMC3970020

[28] Yang B, Liu H, Shi W, Wang Z, Sun S, Zhang G et al Blocking transforming growth factor-β signaling pathway augments antitumor effect of adoptive NK-92 cell therapy. Int Immunopharmacol. 2013 10;17(2):198–204 doi: 10.1016/j.intimp.2013.06.003 23806302 10.1016/j.intimp.2013.06.003

[29] Yoon J-H, Jung SM, Park SH, Kato M, Yamashita T, Lee I et al Activin receptor-like kinase 5 inhibition suppresses mouse melanoma by ubiquitin degradation of Smad4, thereby derepressing eomesodermin in cytotoxic T lymphocytes. EMBO Mol Med. 2013 11;5(11):1720–39 doi: 10.1002/emmm.201302524 24127404 10.1002/emmm.201302524 PMC3840488

[30] Herbertz S, Sawyer JS, Stauber AJ, Gueorguieva I, Driscoll KE, Estrem ST et al Clinical development of galunisertib (LY2157299 monohydrate), a small molecule inhibitor of transforming growth factor-beta signaling pathway. Drug Des Devel Ther. 2015 8 10;9:4479–99 doi: 10.2147/DDDT.S86621 26309397 10.2147/DDDT.S86621 PMC4539082

[31] Park C-Y, Kim D-K, Sheen YY. EW-7203, a novel small molecule inhibitor of transforming growth factor-β (TGF-β) type I receptor/activin receptor-like kinase-5, blocks TGF-β1-mediated epithelial-to-mesenchymal transition in mammary epithelial cells. Cancer Sci. 2011 10;102(10):1889–96 doi: 10.1111/j.1349-7006.2011.02014.x 21707864 10.1111/j.1349-7006.2011.02014.x PMC11158462

[32] Connolly EC, Freimuth J, Akhurst RJ. Complexities of TGF-β targeted cancer therapy. Int J Biol Sci. 2012;8(7):964–78 doi: 10.7150/ijbs.4564 22811618 10.7150/ijbs.4564 PMC3399319

