# Correction: Inhibiting TGF-beta signaling preserves the function of highly activated, in vitro expanded natural killer cells in AML and colon cancer models

**DOI:** 10.1371/journal.pone.0197008

**Published:** 2018-05-02

**Authors:** Folashade Otegbeye, Evelyn Ojo, Stephen Moreton, Nathan Mackowski, Dean A. Lee, Marcos de Lima, David N. Wald

The first two authors, Folashade Otegbeye and Evelyn Ojo, should be noted as contributing equally to this work.

The following information is missing from the Funding section: This study was supported by NIH grant T32 GM007250 to EO.
