# Supplementary material for: Inhibiting TGF-beta signaling preserves the function of highly activated, in vitro expanded natural killer cells in AML and colon cancer models
Source: PLoS One. 2018 Jan 17;13(1):e0191358. doi: 10.1371/journal.pone.0191358 (PMC5771627; doi:10.1371/journal.pone.0191358)
Supplement: S1 Table — (PDF) [file pone.0191358.s001.pdf]

S1 Table. Tables comparing cytotoxic activity of fresh, IL2 activated versus feeder-expanded NK cells

S1A. NK Cell Killing Fresh (IL-2 activated) vs Expanded

NK : HCT116	Percentage of HCT116 cells killed in 4 hours assay			
	Fresh		Expanded	
	A	B	A	B
1:2	-4.2%	7.2%	16.2%	31.3%
1:1	7.8%	2.3%	35.1%	46.7%
2:1	22%	22.5%	42%	57.6%
5:1	56.4%	53.6%	76.2%	76.5%
10:1	87.1%	89.6%	88.5%	87%

S1B. NK Cell Killing Fresh (IL-2 activated) vs Expanded against HT29 cells at 1NK to 1HT29 ratio

	Percentage of HT29 cells killed in 4 hours assay					
	Fresh			Expanded		
	A	B	C	A	B	C
Donor 1	18.8%	28.6%	24.9%	45.45%	58.9%	61.6%
Donor 2	20.3%	3.6%	5.9%	81.8%	86.3%	82.4%
Donor 3	19.8%	19.6%	25.1%	71.4%	71.9%	69.9%

S1C. NK Cell Killing Fresh (IL-2 activated) vs Expanded against HT29 cells at 4NK to 1HT29 ratio

	Percentage of HT29 cells killed in 4 hours assay					
	Fresh			Expanded		
	A	B	C	A	B	C
Donor 1	36.4%	31.1%	24.9%	94.6%	94%	93.6%
Donor 2	38.6%	37.9%	33.9%	94.6%	95.7%	95.2%
Donor 3	29.2%	39.7%	34.8%	92.5%	94.5%	93.2%
