# Supplementary material for: Inhibiting TGF-beta signaling preserves the function of highly activated, in vitro expanded natural killer cells in AML and colon cancer models
Source: PLoS One. 2018 Jan 17;13(1):e0191358. doi: 10.1371/journal.pone.0191358 (PMC5771627; doi:10.1371/journal.pone.0191358)
Supplement: S2 Table — (PDF) [file pone.0191358.s002.pdf]

S2 Table. Tables showing functional analysis of NK cells following exposure to TGF-beta 1 ligand

S2A. Percentage killing of HL60 cells by expanded NK cells at 4NK:1HL60 ratio after 24h and 96h exposure to TGF-beta 1 ligand (5ng/ml and 10ng/ml doses). Triplicate assays

	24 hours			96 hours		
	A	B	C	A	B	C
NK (untreated)	86.7%	85.3%	90.4%	76.4%	72.7%	73.8%
NK + TGF 5ng/ml	77%	75.6%	77%	21%	15.9%	21.9%
NK + TGF 10ng/ml	75.7%	72.9%	79.2%	15.9%	12.9%	15.9%

S2B-2C. Proportion of NK cells expressing NKG2D and CD16 after 24 and 96h exposure to TGF-beta 1 ligand (5ng/mL and 10ng/ml doses)

	24 hours		96 hours	
	NKG2D	CD16	NKG2D	CD16
NK (untreated)	80%	61%	80%	80%
NK + TGF 5ng/ml	29%	59%	28%	35%
NK + TGF 10ng/ml	28%	59%	26%	30%
