# Supplementary material for: Inhibiting TGF-beta signaling preserves the function of highly activated, in vitro expanded natural killer cells in AML and colon cancer models
Source: PLoS One. 2018 Jan 17;13(1):e0191358. doi: 10.1371/journal.pone.0191358 (PMC5771627; doi:10.1371/journal.pone.0191358)
Supplement: S3 Table — (PDF) [file pone.0191358.s003.pdf]

S3 Table. Functional analysis of NK cells in vitro with or without inhibition of TGF beta signaling

S3A-D Table. Mean fluorescence intensity (MFI) of NKG2D and CD16 on NK cells following 24h and 72h exposure to TGF-beta 5ng/ml (T) +/- LY2157299 (G)

		24 hours			72 hours		
		Donor 1	Donor 2	Donor 3	Donor 1	Donor 2	Donor 3
NKG2D	NK control	12840	17477	21074	12720	12688	22965
	NK + T	5910	8064	9808	4920	4369	6337
	NK + T + G	11460	16868	17216	9120	12087	13640
CD16	NK control	29435	36674	39447	34633	41446	41488
	NK + T	22562	33000	39400	19703	30919	36804
	NK + T+ G	25562	34391	39003	29013	43255	46331

S3 E-F Table. Cytotoxicity of expanded NK cells against HT29 cells with or without exposure to TGF-beta ligand 5ng/ml (T) and/or LY2157299 (G). 1NK: 1 HT29. Triplicate assays (A-C)

		24 hours			72 hours		
		A	B	C	A	B	C
Donor 1	NK	45%	59%	62%	55%	58%	58%
	NK + T	11%	21%	12%	4%	14%	22%
	NK + T + G	54%	56%	53%	62%	65%	69%
	NK + G	51%	59%	59%	60%	65%	64%
Donor 2	NK	82%	86%	82%	95%	95%	96%
	NK + T	37%	40%	48%	85%	85%	87%
	NK + T + G	86%	85%	88%	93%	94%	95%
	NK + G	76%	80%	83%	95%	96%	95%
Donor 3	NK	71%	72%	70%	42%	53%	47%
	NK + T	14%	30%	44%	16%	24%	30%
	NK + T + G	85%	88%	89%	70%	78%	78%
	NK + G	76%	79%	79%	61%	59%	68%

S 3G-H Table. Cytotoxicity of expanded NK cells against HCT116 cells with or without exposure to TGF-beta ligand 5ng/ml (T) and/or LY2157299 (G). 1NK: 1 HCT116. Triplicate assays (A-C)

		24 hours			72 hours		
		A	B	C	A	B	C
Donor 1	NK	38%	48%	48%	18%	15%	21%
	NK + T	35%	42%	46%	-1%	5%	15%
	NK + T + G	44%	51%	54%	16%	18%	23%
	NK + G	46%	52%	54%	15%	20%	23%
Donor 2	NK	55%	61%	62%	70%	73%	74%
	NK + T	24%	30%	31%	59%	65%	67%
	NK + T + G	62%	61%	69%	74%	78%	79%
	NK + G	57%	75%	60%	70%	72%	78%
Donor 3	NK	64%	68%	70%	28%	27%	28%
	NK + T	56%	59%	59%	18%	25%	18%
	NK + T + G	81%	78%	83%	30%	32%	32%
	NK + G	65%	72%	69%	35%	31%	22%

S 3I. Cytotoxicity of expanded NK cells against HL60 cells with or without 72 hours exposure to TGF-beta ligand 5ng/ml (T) and/or LY2157299 (G). Triplicate assays (A-C) per donor; at 1:1 and 4:1 ratios (NK to HL60)

		Donor 1			Donor 2		
		A	B	C	A	B	C
1NK:1HL60	NK	69%	71%	72%	23%	30%	36%
	NK + T	29%	33%	36%	10%	13%	10%
	NK + T + G	69%	71%	72%	37%	35%	38%
	NK + G	74%	76%	74%	31%	33%	36%
4NK:1HL60	NK	80%	81%	80%	47%	42%	44%
	NK + T	42%	46%	49%	24%	22%	26%
	NK + T + G	62%	61%	69%	46%	50%	56%
	NK + G	80%	83%	83%	45%	52%	47%
