# Supplementary material for: Inhibiting TGF-beta signaling preserves the function of highly activated, in vitro expanded natural killer cells in AML and colon cancer models
Source: PLoS One. 2018 Jan 17;13(1):e0191358. doi: 10.1371/journal.pone.0191358 (PMC5771627; doi:10.1371/journal.pone.0191358)
Supplement: S4 Table — (PDF) [file pone.0191358.s004.pdf]

S4 Table. Comparisons of TNF-alpha, IFN-gamma, Perforin and Granzyme release

S4A-B. ELISA assay of TNF-alpha and IFN-gamma in supernatants of NK cells co-incubated for 4 hours with HT29 cells at 40,000 NK + 10,000 HT29 cells/ well; 96-well plate; triplicate wells

	TNF-alpha (pg/ml)			IFN-gamma (pg/ml)		
	A	B	C	A	B	C
NK	26.5	28.456	26.935	41.483	45.621	38.724
NK + T	15.84783	18.456	15.196	29.414	33.896	38.379
NK + T + G	32.587	31.282	38.674	48.379	42.862	42.517
NK + G	29.761	28.674	30.196	46.310	43.896	44.931

S4C-D. ELISA assay of Perforin and Granzyme B in supernatants of NK cells co-incubated for 2 hours with HL60 cells at 1,000,000 NK + 1,000,000 HL60 cells/ well; 12-well plate; triplicate wells

	Perforin (ng/ml)			Granzyme B (ng/ml)		
	A	B	C	A	B	C
NK	12.648	13.224	12.887	1.765	1.788	1.824
NK + T	5.399	5.647	5.516	1.179	1.125	1.098
NK + T + G	11.525	11.622	11.7	1.809	1.789	1.782
NK + G	12.695	12.75	13.0	1.823	1.811	1.825
