# Supplementary material for: Inhibiting TGF-beta signaling preserves the function of highly activated, in vitro expanded natural killer cells in AML and colon cancer models
Source: PLoS One. 2018 Jan 17;13(1):e0191358. doi: 10.1371/journal.pone.0191358 (PMC5771627; doi:10.1371/journal.pone.0191358)

S1 Fig. Representative images of colon cancer metastasis xenograft showing CD45 IHC of liver FFPE sections staining for human NK cells. 10X view of slide sections; slide capture using VENTANA digital imaging software

S1 FigA. Vehicle only group; all sections showing tumor infiltration; no CD45+ cells observed by IHC

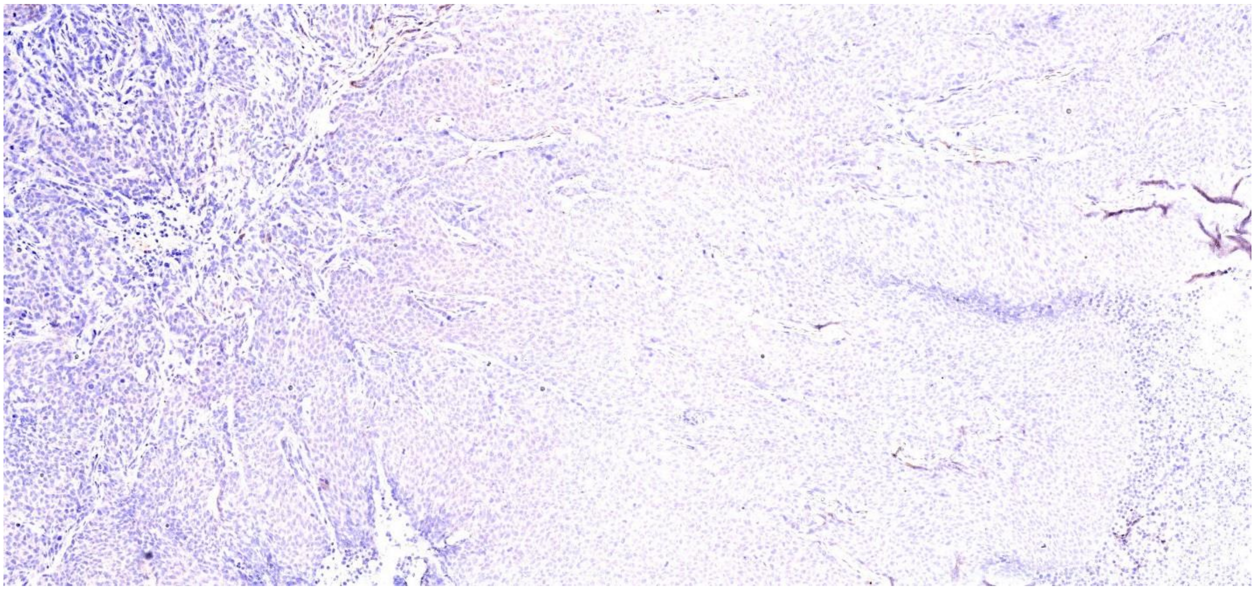

S1 FigB. LY2157299 (Galunisertib) only group; all sections showing tumor infiltration; no CD45+ cells observed by IHC

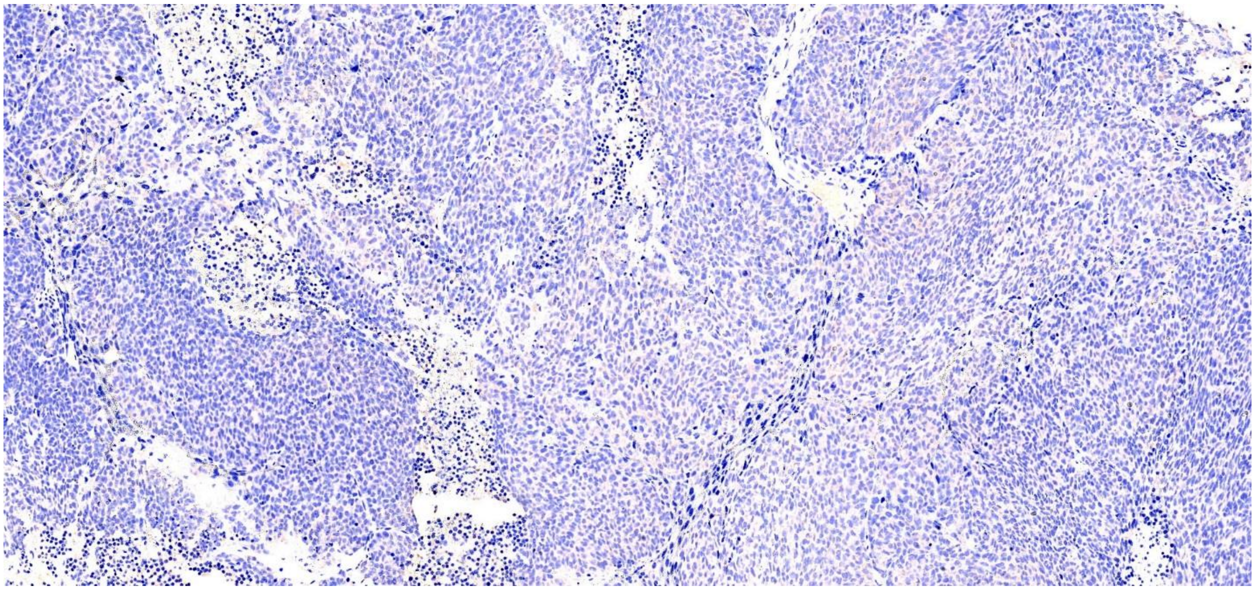

S1 FigC. NK cell only group showing section of tumor involvement with infiltrating NK cells (CD45+ brown cells)

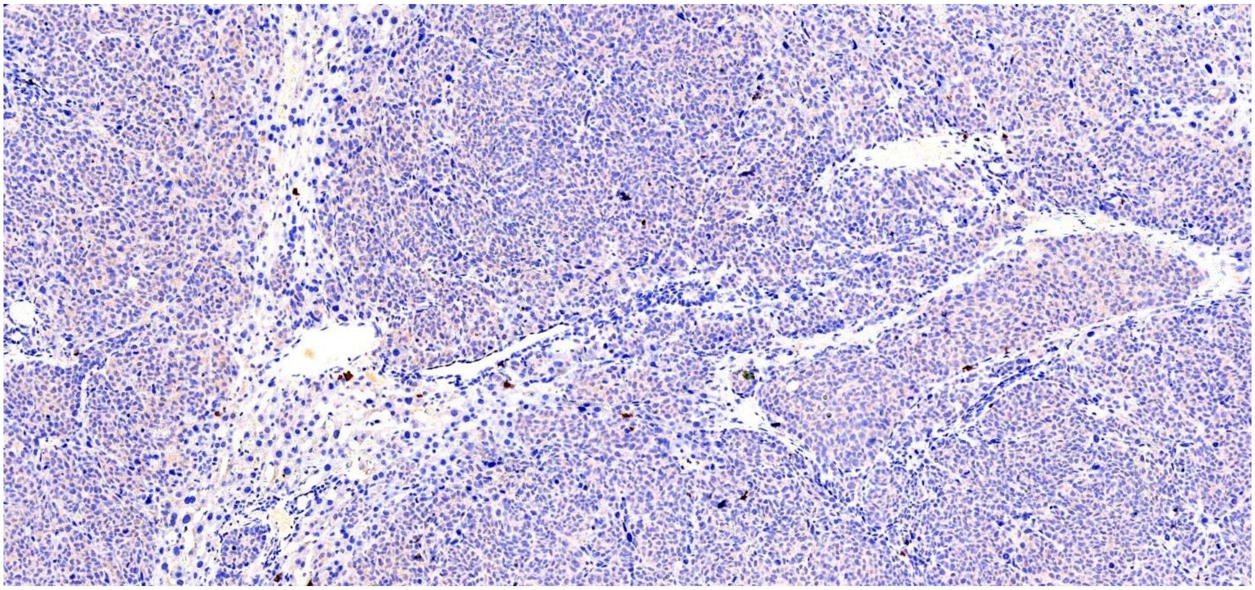

S1 FigD. NK Cells plus LY2157299 group showing section with rare residual tumor showing surrounding infiltrating NK cells (CD45+ brown cells) in normal liver tissue

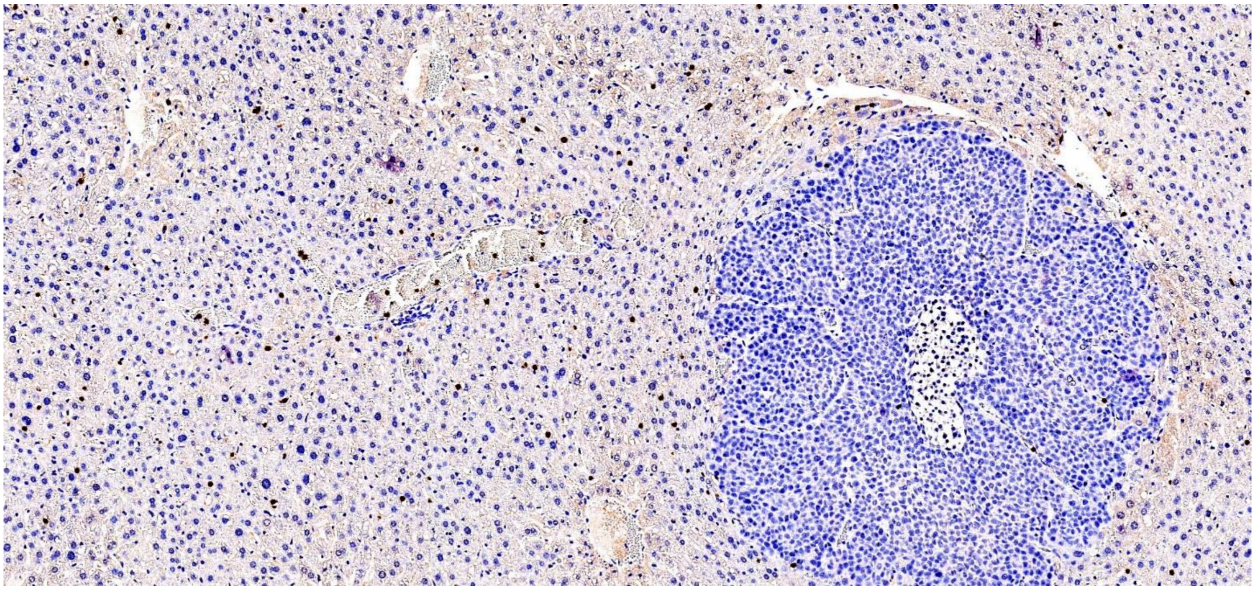

Supplement: S1 Fig — (PDF) [file pone.0191358.s005.pdf]
