# Supplementary material for: Inhibiting TGF-beta signaling preserves the function of highly activated, in vitro expanded natural killer cells in AML and colon cancer models
Source: PLoS One. 2018 Jan 17;13(1):e0191358. doi: 10.1371/journal.pone.0191358 (PMC5771627; doi:10.1371/journal.pone.0191358)
Supplement: S2 Fig — (PDF) [file pone.0191358.s006.pdf]

S2 Fig. Dose titration of LY2157299 for preservation of NK cell cytotoxic function. Using NK cell to HCT116 ratios of 1:1 and 4:1; 4h co-culture cytotoxicity assay in triplicates. LY2157299 (G) doses of 5uM, 2.5uM, 1uM and 0.5uM

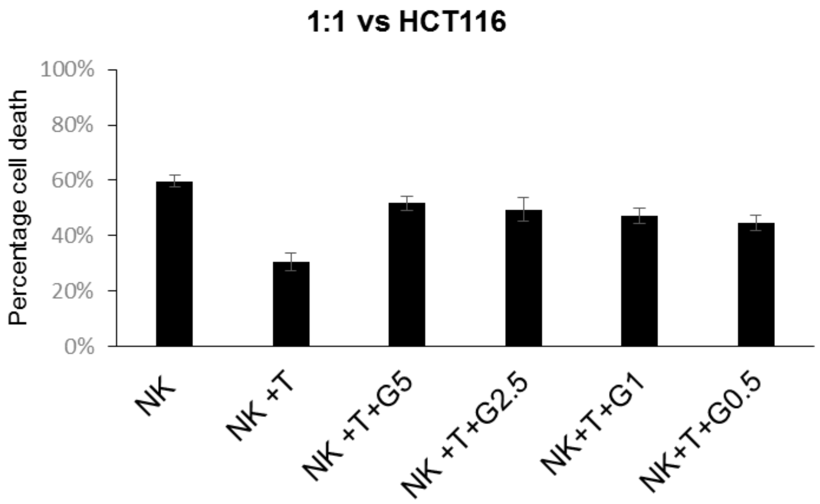

2-tailed T test	p-value
G5 vs G0.5	0.0099
G5 vs G1	0.044
G5 vs G2.5	0.403

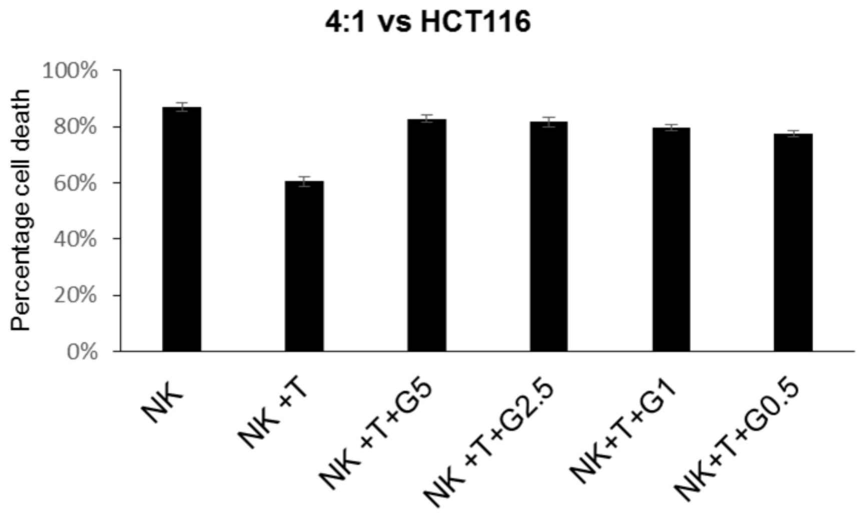

2-tailed T test	p-value
G5 vs G0.5	0.0009
G5 vs G1	0.0081
G5 vs G2.5	0.3362
